# Highly Transparent and Surface-Plasmon-Enhanced Visible-Photodetector Based on Zinc Oxide Thin-Film Transistors with Heterojunction Structure

**DOI:** 10.3390/ma12213639

**Published:** 2019-11-05

**Authors:** Cheng-Jyun Wang, Hsin-Chiang You, Kuan Lin, Jen-Hung Ou, Keng-Hsien Chao, Fu-Hsiang Ko

**Affiliations:** 1 Department of Materials Science and Engineering, National Chiao-Tung University, 1001 University Rd., Hsinchu 30010, Taiwan; cjwang.nano03g@nctu.edu.tw (C.-J.W.); osho8888@gmail.com (K.L.); harveyou@hotmail.com.tw (J.-H.O.); lces32051.nano07g@nctu.edu.tw (K.-H.C.); 2 Department of Electronic Engineering, National Chin-Yi University of Technology, No. 57, Sec. 2, Zhongshan Rd., Taiping Dist., Taichung 41170, Taiwan; hcyou@ncut.edu.tw

**Keywords:** zinc oxide-based thin-film transistors, gold-nanoparticles, phototransistors, spray pyrolysis, plasmonic energy detection

## Abstract

Highly transparent zinc oxide (ZnO)-based thin-film transistors (TFTs) with gold nanoparticles (AuNPs) capable of detecting visible light were fabricated through spray pyrolysis on a fluorine-doped tin oxide substrate. The spray-deposited channel layer of ZnO had a thickness of approximately 15 nm, and the thickness exhibited a linear increase with an increasing number of sprays. Furthermore, the ZnO thin-film exhibited a markedly smoother channel layer with a significantly lower surface roughness of 1.84 nm when the substrate was 20 cm from the spray nozzle compared with when it was 10 cm away. Finally, a ZnO and Au-NP heterojunction nanohybrid structure using plasmonic energy detection as an electrical signal, constitutes an ideal combination for a visible-light photodetector. The ZnO-based TFTs convert localized surface plasmon energy into an electrical signal, thereby extending the wide band-gap of materials used for photodetectors to achieve visible-light wavelength detection. The photo-transistors demonstrate an elevated on-current with an increase of the AuNP density in the concentration of 1.26, 12.6, and 126 pM and reach values of 3.75, 5.18, and 9.79 × 10^−7^ A with applied gate and drain voltages. Moreover, the threshold voltage (Vth) also drifts to negative values as the AuNP density increases.

## 1. Introduction

Thin-film transistors (TFTs) based on transparent conductive oxide (TCO) materials have attracted increasing attention for their applications in optoelectronic devices and ultraviolet (UV) light detectors in the past few years, due to their advantages including a wide-band gap, suitable optical transparency, simple solution-based manufacturing, and low-temperature processability [1,2,3,4]. Solution-based processing of TCO materials, such as zinc oxide (ZnO), Ga_2_O_3_ and SnO_2_ have been extensively studied for application in the channel layer and ultraviolet (UV) optical band detecting layer [5,6]. ZnO is a suitable material for use in the production of UV sensitizers and broad band transparent conductors for visible-blind UV detection with the additional advantages of high transparency; for direct band-gap semiconductors with band-gap of 3.37 eV and the solution processability enables high-performance photo-detecting devices to be manufactured simply, at lower-cost, and with large-areas [7,8,9,10,11,12]. Therefore, TCO materials are considered the most appropriate semiconducting materials because of their transparency, high-performance electrical, and UV light detecting properties. These materials have been used a photodetecting layer, which can convert a UV wavelength region into an electrical signal. An intrinsically wide band-gap limits the light absorption of a high-energy UV region. Several methods have been reported to improve the photo-responsivity of TCO-based TFTs in a visible wavelength region, such as the use of quantum dots, and polymeric absorption thin-film layers [12,13,14]. In particular, the metallic nanoparticles of gold nanoparticles (AuNPs) exhibit distinct surface plasmonic properties because of the abundance of energized electrons on the particle surfaces [15]. The metallic nanoparticles (NPs) of localized surface plasmon resonance (LSPR), which is an efficient conversion of plasmon energy into an electrical signal for an illumination-specific light wavelength, can be employed for visible-light photodetection. The ZnO and AuNPs heterojunction nanohybrid structure enables plasmonic energy detection as a form of an electrical signal and constitutes an ideal combination of visible light photodetection. Photons absorbed in a metal nanostructure generate hot electrons which can cross by thermionic diffusion at the boundary between metal and a semiconducting layer, thereby generating a photocurrent or modifying the energy band gap in the semiconducting layer of ZnO [16,17,18,19]. Therefore, this phenomenon is promising for the development of a photodetector with TCO-based TFTs and increased visible-light detection wavelengths.

In recent years, solution-based manufacturing technologies have been promising candidates for the manufacturing of next generation materials for sensing electronic devices due to their potential benefits including reduced production costs, large area fabrication, low temperature processing, and suitability for various substrates e.g., flexible and glass substrates [20,21,22,23,24,25]. Generally, the spin-coating, ink-jet printing, and spray pyrolysis deposition are the prevalently used methods for manufacturing the channel layer of TFTs using solution-based technologies [26,27,28,29,30]. Although a more uniform and ultra-thin film is generated in spin-coating and numerous studies have employed spin coating, it is not a suitable method for large-area manufacturing, particularly for glass substrates and products in the electronic display. Among the various solution-based deposition techniques, spray pyrolysis is unique because it combines the synthetic versatility and large-area manufacturing of solution processing by using favorable growth mechanics, thus generating a film with extraordinary electrical characteristics [31]. Furthermore, this deposition method is used as a scalable technology for depositing TCO materials in numerous applications, including semiconducting industry and solar cells [32,33,34,35,36]. In particularly, this depositing method can spray materials over a large-area on glass substrates.

## 2. Materials and Methods

**Figure 1:**
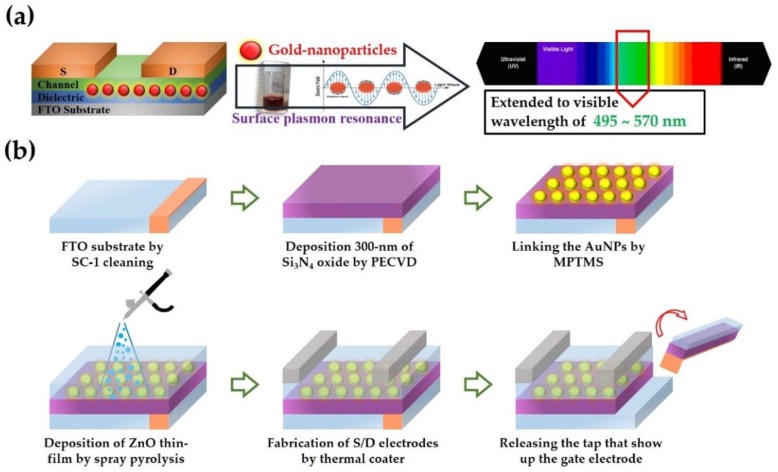
Photodetector with ZnO-based thin-film transistors (TFTs) and gold nanoparticles (AuNPs) fabricated on a transparent fluorine-doped tin oxide (FTO) structure. (**a**) Schematic and illumination of the photodetector. (**b**) Fabrication procedure of the photodetector with ZnO-based TFTs through spray pyrolysis processing. Plasma-enhanced chemical vapor deposition (PECVD).

Figure 1a presents the zinc oxide-based thin-film transistors (ZnO-based TFTs) with gold nanoparticles (AuNPs) structure for extending the range of light absorbed. First, the transparent conducting oxide solution of ZnO was prepared by dissolving zinc acetate-dihydrate [Zn(CH_3_COO)_2_·2H_2_O] into ethanol (CH_3_CH_2_OH, absolute ≥99.8%) as a precursor with a concentration of 0.05 M, and the solution was then gently stirred for 1.5 h at 60 °C until a highly transparent solution was obtained. The gold-nanoparticle (AuNP) solution was prepared by adding 1 mL of 38.8 mL aqueous trisodium citric acid (Na_3_C_6_H_5_O_7_, TSC) into 1 mM of aqueous boiling solution of hydrogen tetrachloroaurate (HAuCl_4_), and the resulting mixture was continuously boiled for 30 min until a red solution was obtained. Then, AuNPs with a diameter of 13 nm were obtained.

For device fabrication, a transparent, conducting fluorine-doped tin oxide (FTO) substrate was treated via a standard clean one (SC-1) cleaning procedure to eliminate organic and particle contamination before depositing an oxide film. Then, a 300 nm thick layer of silicon nitride oxide (Si_3_N_4_) was deposited on the FTO substrate through the low-temperature processing of a plasma-enhanced chemical vapor deposition (PECVD) system at 300 °C. Before incorporating AuNP nanostructures, electrical characteristics were evaluated for all fabricated the ZnO-based TFT devices. The ability for plasmonic energy detection included incorporating AuNPs in an active channel layer of ZnO, thus fabricating a metal-semiconducting junction structure. The AuNPs were connected with the ZnO channel layer and silicon nitride oxide by using (3-mercaptopropyl)trimethoxysilane (MPTMS) with concentrations of 1.26, 12.6, and 126 pM. Subsequently, the ZnO precursor solution was then spray deposited according to the various spray pyrolysis deposition conditions of different layers, such as the distance between the substrate and spray nozzle and active material used for the n-type semiconducting and photosensitive layer, followed by air annealing at 300 °C for 1 hour. Finally, the source and drain electrode regions were defined by metal mask followed by a thermal coater deposition of 300 nm thick aluminum. The thin-film transistors of channel length and width were 70 μm and 2000 μm, respectively, as shown in Figure 1b.

## 3. Results and Discussion

Spray pyrolysis is a unique depositing method which can spray materials over a large area on a glass substrate. Bo-Xuan Yang et al. presented a transparent ZnO-based thin film transistors that were fabricated on a glass substrate with solution processes. The TFTs were indeed highly transparent (transmission of ~70% in the visible spectrum) and showed excellent electrical characteristics including an I_ON_/I_OFF_ ratio of approximately 10^4^ through the spray pyrolysis to deposit the active ZnO channel layer. These results represent significant achievements toward low cost and low energy consumption transparent oxide TFTs based on simple solution-based processes [24,37]. However, the spray-deposited film still exhibited high roughness of the micro-grade film as compared with the spin-coated film, and a high temperature manufacturing process >400 °C was involved.

**Figure 2:**
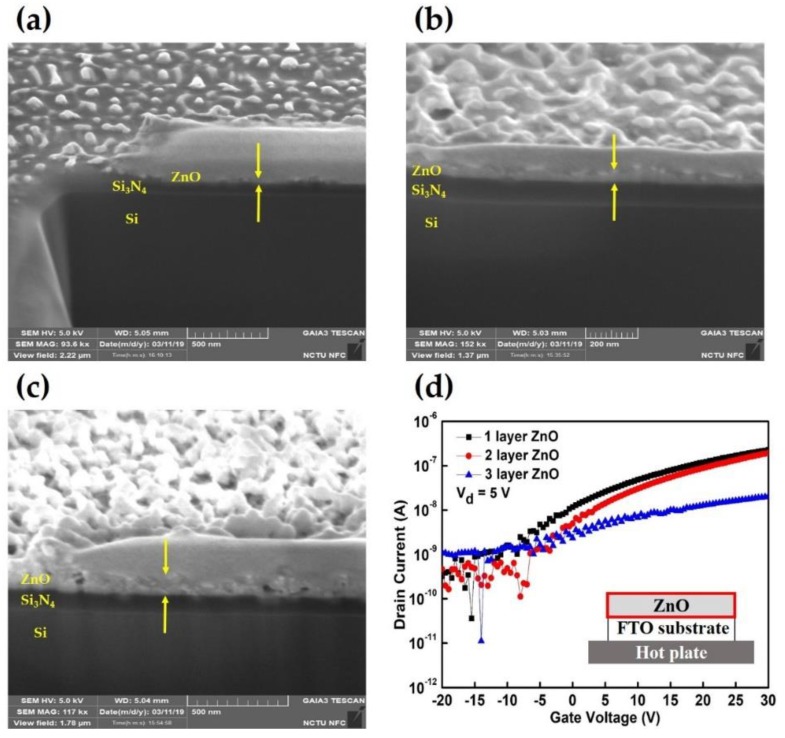
Focus ion beam cross-section images of the ZnO-based TFTs with the ZnO channel layer sprayed (**a**) once, (**b**) twice, and (**c**) thrice; (**d**) typical transfer characteristics of drain-source current versus gate voltage (I_DS_-V_GS_) of ZnO-based TFTs with ZnO channel layers that have thicknesses of 15.98, 28.54, and 58.90 nm.

Herein, we established a simple, low-temperature manufacturing (all processes were about 300 °C) and novel structure of highly transparent (>75% in the visible-light region between 400 and 800 nm wavelength) of the ZnO-based TFTs with AuNPs heterojunction which were fabricated on a FTO structure for extending the light absorption wavelength during visible-light detection. These TCO materials can convert a UV wavelength region into an electrical signal. Moreover, the ZnO thin-film exhibited a markedly smoother channel layer with significantly lower surface roughness of 1.84 nm via the spray-deposited method, and superior electrical performance with higher electron mobility in a linear region and a higher I_ON_-I_OFF_ ratio of approximately 10^5^. These electrical characteristics are not only competitive compared to the previous reports but also compared to those of the ZnO-based photo-detectors via spray pyrolysis. To validate the TFT photodetection ability and photosensitivity in this study, the morphology and typical transfer electrical characteristics of the TFT fabricated through spray deposition under various conditions were evaluated. Figure 2a–c respectively present the cross-sectional images of spray deposition with one, two, and three layers of 0.05 M ZnO thin-film through post-annealing at 300 °C. The semiconducting channel with ZnO thin film thickness of approximately 15.98, 28.54, and 58.90 nm were obtained through one, two, and three rounds of spray deposition, respectively. The thickness of the spray-deposited layer was approximately 15 nm, and it linearly increased with the increasing number of sprays. This observation indicates that the spray deposition technique can provide a stable and large-scale approach for depositing a semiconducting ZnO channel layer that is inexpensive and is easy to handle for the fabrication of micro-electronics and large-scale film manufacturing. To evaluate the electrical characteristics of the ZnO-based TFTs with channel layers of different thickness, the drain-current (I_DS_) was first controlled before measuring the electrical properties of the devices by applying a back-gate bias voltage (V_GS_) to the FTO substrate used as a back-gate electrode. Figure 2d shows the typical transfer characteristics of drain-source current versus gate voltage (I_DS_-V_GS_) of the ZnO-based TFTs with ZnO channel layers that have thicknesses of 15.98, 28.54, and 58.90 nm, and which were operated by varying V_GS_ from –20 to 30 V at a constant V_DS_ of 5 V on a logarithmic scale. Both devices demonstrated saturation behaviors when V_GS_ became more positive, and the devices’ properties were switched on by the electrons accumulated in the n-type ZnO channel layer under a positive V_GS_, which was applied by the back gate electrode of the FTO substrate. When the V_GS_ was 30 V, the on-off current (I_ON_-I_OFF_) ratio was approximately 10^3^, and the device with a ZnO channel layer with a thickness of 58.90 nm exhibited an unsatisfactory I_ON_-I_OFF_ ratio of approximately 10^1^ because the thickness of the ZnO layer was too great for the V_DS_ to be sufficient to turn on the channel properties.

**Figure 3:**
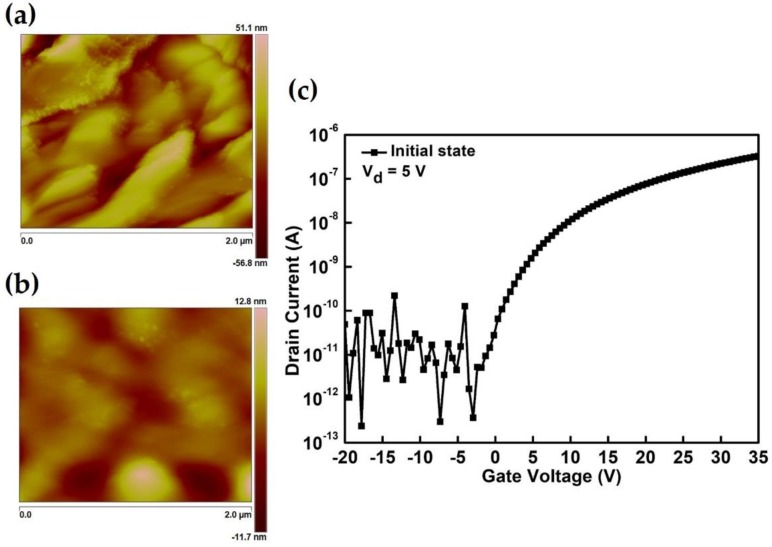
Atomic force microscopy images of the semiconducting channel ZnO thin film spray-deposited at the distances of (**a**) 10 cm and (**b**) 20 cm between the spray nozzle and substrate. (**c**) Transfer characteristics of drain-source current versus gate voltage (I_DS_-V_GS_) of two layers of several semiconducting channel ZnO thin films deposited at a distance of 20 cm between the nozzle and substrate.

Furthermore, the roughness of the interaction between the spray nozzle and the substrate was investigated at different distances. Continuous ZnO channel layers were deposited through sequential, continual spray coatings with two runs (two spraying cycles); each spraying cycle lasted for 10 seconds. Figure 3 shows the effect of spray deposition at different distances on the surface topography of the ZnO thin film layer and TFT electrical performance. Figure 3a,b respectively present the atomic force microscopy (AFM) images of the dried ZnO thin film patterns of individual spray-deposited layers on Si/Si_3_N_4_ substrates at the distances of 10 and 20 cm. The results reveal that the average roughness of the ZnO thin film deposition at the distances of 10 and 20 cm between the nozzle and substrate were 11.0 and 1.84 nm, respectively. The ZnO thin film morphology at the distance of 20 cm exhibited a significantly smoother channel layer with significantly lower surface roughness compare with the ZnO thin film at 10 cm. This is a crucial observation because the increasing distance of the spray nozzle and substrate can suppress the impact of the valve pressure on the ZnO thin film, and a smoother semiconducting layer was created for the reliable electrical operation of electronic devices through spray pyrolysis deposition. Figure 3c presents the typical transfer characteristics of drain-source current versus gate voltage (I_DS_-V_GS_) for two layers of several semiconducting channel ZnO thin films deposited when the distance between the nozzle and substrate was 20 cm. The aforementioned ZnO surface topography discussion indicates that TFTs with ZnO thin film spray-deposited at 20 cm exhibited a superior electrical performance with higher electron mobility in the linear region and a higher I_ON_-I_OFF_ of approximately 10^5^. Furthermore, Figure S1 presents the 10 devices of typical transfer characteristics of drain-source current versus gate voltage (I_DS_-V_GS_) and the square root of the drain current-gate voltage transfer characteristics curves, and our films exhibited superior stable electrical performance with a lower leakage current and higher electron mobility in the linear region. This clearly demonstrates the benefits of a spray-deposition ZnO channel film at a distance of 20 cm.

**Figure 4:**
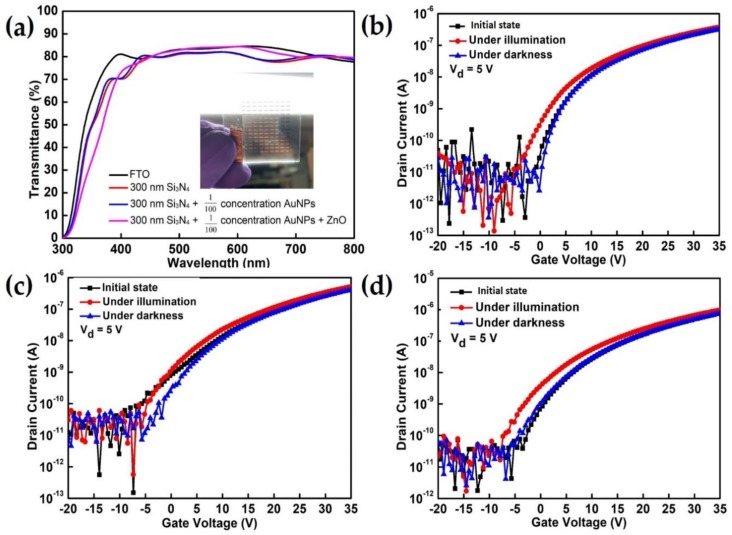
(**a**) Transmittance of the phototransistor based on ZnO and AuNPs on the transparent glass of an FTO substrate (inset image shows the optical photo on our devices). Typical transfer characteristics of the ZnO/AuNPs-based structure TFTs with and without visible-light illumination, where AuNPs were placed between the semiconducting channel ZnO and Si_3_N_4_ gate dielectric with concentrations of (**b**) 1.26, (**c**) 12.6, and (**d**) 126 pM.

The transparent photodetector with ZnO-based TFTs and AuNP structure was fabricated on a glass substrate of FTO, as shown in Figure 4a. The device exhibits outstanding transparency with high transmittance (>75% in the visible-light region between 400 and 800 nm wavelength). As seen in the inset image of Figure 4a, visible-light can penetrate our devices, and can penetrate an active channel consisting of a photosensitive region with ZnO and AuNPs of 20 nm thickness, where AuNPs were placed between the semiconducting channel ZnO and Si_3_N_4_ gate dielectric with concentrations of 1.26, 12.6, and 126 pM, to validate the photosensitivity of an active ZnO thin film with different concentrations of AuNPs for visible-light illumination (under halogen lamp illumination). Figure S2 displays a scanning electron microscopy image of AuNPs distributed in the channel region of ZnO between the source and drain electrodes. 

Recently, Hojoong Choi et al. have reported a solution-processed ZnO/SnO_2_ bilayer ultraviolet photo-transistor device with a high responsivity of ~82.28 A/W and fast photo-response and recovery times of 5.39 and 4.37 seconds, respectively, under the wavelength of 365 nm UV illumination [8]. This is a perfect structure and approach for improving the performance of UV photo-transistors. However, the ZnO can convert only the UV wavelength region into an electrical signal because the intrinsically wide band-gap limits the light absorption of the high-energy UV region. In contrast, for both devices in this study, the on-current was significantly enhanced under visible light wavelength by the incorporation of AuNPs in the ZnO semiconducting channel layer, and the photo-current increased with the increasing AuNP concentrations when the amplification interval of the saturation region of the on-current between the gate voltage of 30–35 V, as indicated in Figure S3. The typical transfer I_DS_-V_GS_ characteristics of the initial state of the devices, with and without visible-light illumination, are presented in Figure 4b–d. Photo-transistors demonstrated increased on-current values reaching 3.75, 5.18, and 9.79 × 10^−7^ A, with applied gate and drain voltages with increasing AuNP density and concentrations of 1.26, 12.6, and 126 pM. As mentioned, plasmonic energy absorption generates hot electrons in gold NPs and these hot electrons can migrate across the ZnO-AuNPs heterojunction and diffuse in the active ZnO film with a specific light wavelength, thereby increasing the saturation current of devices. Several methods have been reported to improve the photo-responsivity of TCO-based TFTs in a visible wavelength region, such as the use of quantum dots, and polymeric absorption thin-film layers [12,13,14]. However, the nanostructure arrangement within large-area uniformity of quantum dots, and polymeric absorption layers are a critical issue, and the rough film will create poor electrical characteristics. Those material’s sensitivity to visible light wavelengths are still quite low. Compared to the previous reports, we achieved a large-area uniformity of ZnO and Au-NPs heterojunction nanohybrid structure (roughness ~1.8 nm) of photodetectors, that not only amplifies the photo-current but also efficient conversion of plasmon energy into an electrical signal for an illumination-specific light wavelength. As expected, the threshold voltage (V_th_) also drifts to considerably more negative values with increasing AuNP density. Figure S1 presents the square root of the drain current-gate voltage transfer characteristics curves of the initial state of the devices, under illumination and with eliminated light illumination. The △V_th_ were the values 2.6, 3.1, and 3.6 V, which corresponded to the concentrations of 1.26, 12.6, and 126 pM, respectively, under the visible light illumination. In all devices, the electrical parameters of the TFT device were affected by the increasing on-current, and △Vth values became increasingly negative when the AuNPs–ZnO (metal-semiconductor) heterojunction nanohybrids structure was subject to visible to visible-light illumination without any accompanying field-effect mobility (μ_FE_) degradation. The Vth drift into negative values was attributed to the corresponding visible-light wavelength of AuNPs, as well as photo-induced free carriers, which can be vigorously activated in ZnO films, in which localized surface plasmon energy has induced electron-hole pairs in the ZnO film. The electrical characteristics recovered their initial states after the visible-light illumination was turned off. These results indicate that highly transparent phototransistors based on ZnO TFTs with an AuNP structure that are highly photo-sensitive to visible-light wavelength can be fabricated using transparent, conductive oxide materials and metallic nanoparticles with a wide bandgap material.

**Figure 5:**
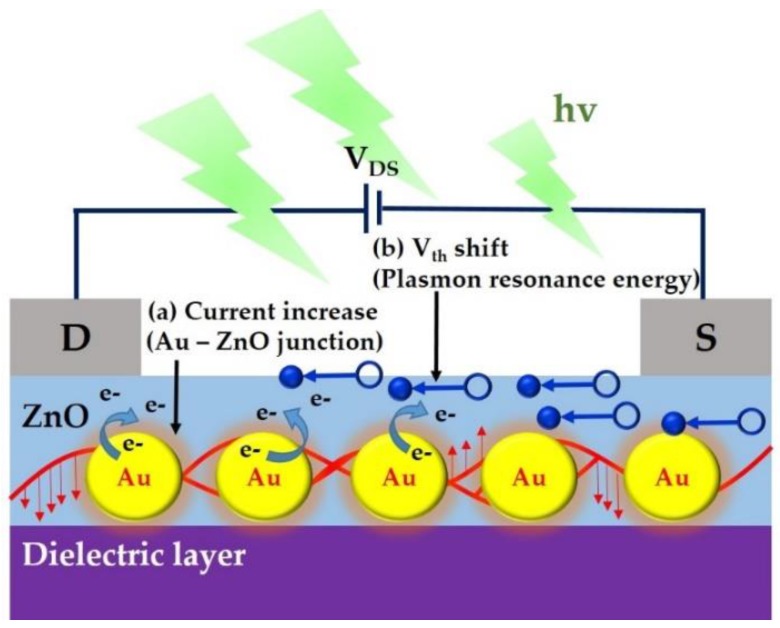
Mechanism of visible-light induced photocurrent and energy-band modification on the hybrid junction of metallic AuNPs and semiconducting ZnO film.

The extension of the light absorbed during visible-light photodetection is obtained through the amplification of plasmon detection and Figure 5 depicts the working principle. A metal-semiconducting junction structure can convert plasmonic energy into an electrical signal and increase the current with a specific visible light wavelength. In this study, the nanoparticles with a diameter of 13 nm exhibited strong plasmonic absorption at light wavelengths of 495–570 nm (green light). Photons absorbed in the metallic particles of a nanostructure that generates hot electrons in junctions in the semiconducting channel layer, thereby generating photo-current (saturation current increasing) and modifying the energy band, in semiconducting ZnO channel properties (Vth drift to negative values). The ZnO-based TFTs collects plasmonically induced hot electrons from the AuNPs, and these hot electrons can migrate across the ZnO-AuNPs heterojunction and diffuse in the active semiconducting channel of ZnO film with a specific light wavelength of 495–570 nm, thereby increasing the saturation current. Moreover, the photo-transistors demonstrated increased on-current with applied gate and drain voltages accompanied by the increasing AuNP density and concentrations of 1.26, 12.6, and 126 pM. Furthermore, the threshold voltage (Vth) also drifted to considerably more negative values with increasing AuNPs density. This was attributed to the corresponding visible-light wavelength of AuNPs, as well as photo-induced free carriers, which can be vigorously activated in a ZnO film, in which localized surface plasmon energy has induced electron-hole pairs in the ZnO film. Therefore, the ZnO/AuNPs-based TFTs converted localized surface plasmon energy into an electrical signal, thus extending the wide band gap of the material used in the photodetector to enable visible-light wavelength detection.

## 4. Conclusions

In summary, highly transparent ZnO-based TFTs with AuNPs were fabricated on a FTO structure, for extending the light absorption wavelength during visible-light detection through the solution-based processing of spray pyrolysis, and a thickness dependent experiment was performed on the ZnO films. The thickness of the deposited spray was approximately 15 nm, and it increased linearly with the increasing number of sprays. This observation indicates that the spray deposition technique can provide a stable approach for large-scale deposition of semiconducting ZnO channel layers. Furthermore, a comparison of the roughness of ZnO thin films deposited at distances of 10 and 20 cm between the nozzle and substrate revealed that the distance of 20 cm for deposition produced a significantly smoother channel layer on the ZnO thin film with lower surface roughness compared with deposition at the distance of 10 cm. Finally, a visible-light photodetector was fabricated using a hybrid active channel material based on ZnO film and AuNPs. A wide-bandgap ZnO film was used as a transparent semiconducting channel layer, and metallic AuNPs were adopted to absorb and induce photocurrent and energy-band modifications under visible-light illumination. A metal-semiconducting junction structure can convert plasmonic energy into an electrical signal and increase the current under a specific visible-light wavelength. Therefore, the results indicate that highly transparent phototransistors based on ZnO TFTs AuNP structures that are highly sensitive to visible-light can be fabricated using TCO materials and metallic nano-particles with wide-bandgaps.

## Supplementary Materials

The following are available online at https://www.mdpi.com/1996-1944/12/21/3639/s1, Figure S1: (a) Transfer characteristics of drain-source current versus gate voltage (IDS-VGS) of 10 devices of several semiconducting channel ZnO thin films deposited at a distance of 20 cm between the nozzle and substrate. (b) The square root of the drain current-gate voltage transfer characteristics curves of the 10 devices, Figure S2: The Scanning electron microscopy (SEM) image of the gold NPs under the ZnO thin-film. The scale bar in the SEM image is 100 nm, Figure S3: The typical transfer IDS-VGS characteristics of amplification intervals for the saturation region of the on-current between gate voltage operation at 30–35 volts, and the devices with AuNPs concentration of (a) 1.26, (b) 12.6, and (c) 126 pM. The square root of the drain current-gate voltage transfer characteristics curves of the devices with initial state, under illumination and without light illumination, the △Vth were 2.6, 3.1, and 3.6 volts which corresponded to the concentrations of (d) 1.26, (e) 12.6, and (f) 126 pM, respectively.
