# Supplementary material for: Highly Transparent and Surface-Plasmon-Enhanced Visible-Photodetector Based on Zinc Oxide Thin-Film Transistors with Heterojunction Structure
Source: Materials (Basel). 2019 Nov 5;12(21):3639. doi: 10.3390/ma12213639 (PMC6862527; doi:10.3390/ma12213639)
Supplement: Supplementary file 1 [file materials-12-03639-s001.pdf]

materials

Supplementary Materials

Highly Transparent and Surface-Plasmon-Enhanced Visible-Photodetector Based on Zinc Oxide Thin-Film Transistors with Heterojunction Structure

Cheng-Jyun Wang [1], Hsin-Chiang You [2], Kuan Lin [1], Jen-Hung Ou [1], Keng-Hsien Chao [1] and Fu-Hsiang Ko [1,*]

[1] Department of Materials Science and Engineering, National Chiao-Tung University, 1001 University Rd., Hsinchu 30010, Taiwan (R.O.C.); cjwang.nano03g@nctu.edu.tw (C.-J.W.); osho8888@gmail.com (K.L.); harveyou@hotmail.com.tw (J.-H.O.); lces32051.nano07g@nctu.edu.tw (K.-H.C.)

[2] Department of Electronic Engineering, National Chin-Yi University of Technology, No. 57, Sec. 2, Zhongshan Rd., Taiping Dist., Taichung 41170, Taiwan (R.O.C.); hcyou@ncut.edu.tw

* Correspondence: fhko@mail.nctu.edu.tw; Tel.: +886-3-5712121

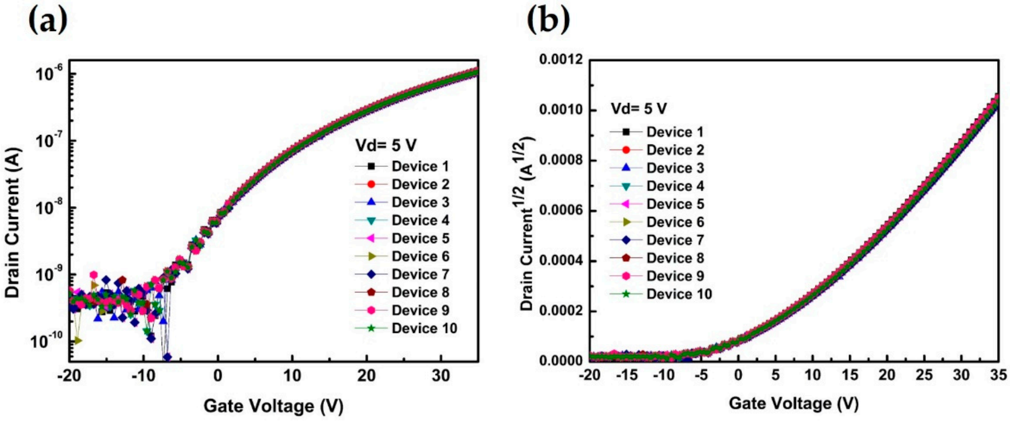

Figure S1. (**a**) Transfer characteristics of drain-source current versus gate voltage (I_{DS}-V_{GS}) of 10 devices of several semiconducting channel ZnO thin films deposited at a distance of 20 cm between the nozzle and substrate. (**b**) The square root of the drain current-gate voltage transfer characteristics curves of the 10 devices.

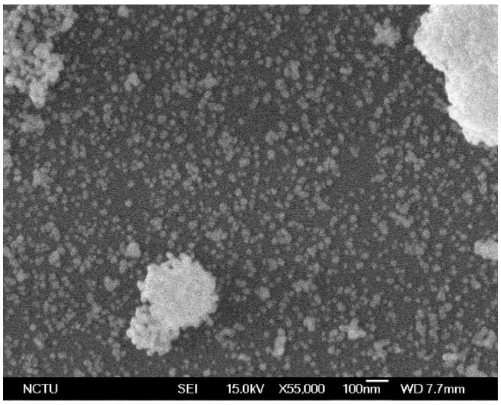

Figure S2. The scanning electron microscopy (SEM) image of the gold NPs under the ZnO thin-film. The scale bar in the SEM image is 100 nm.

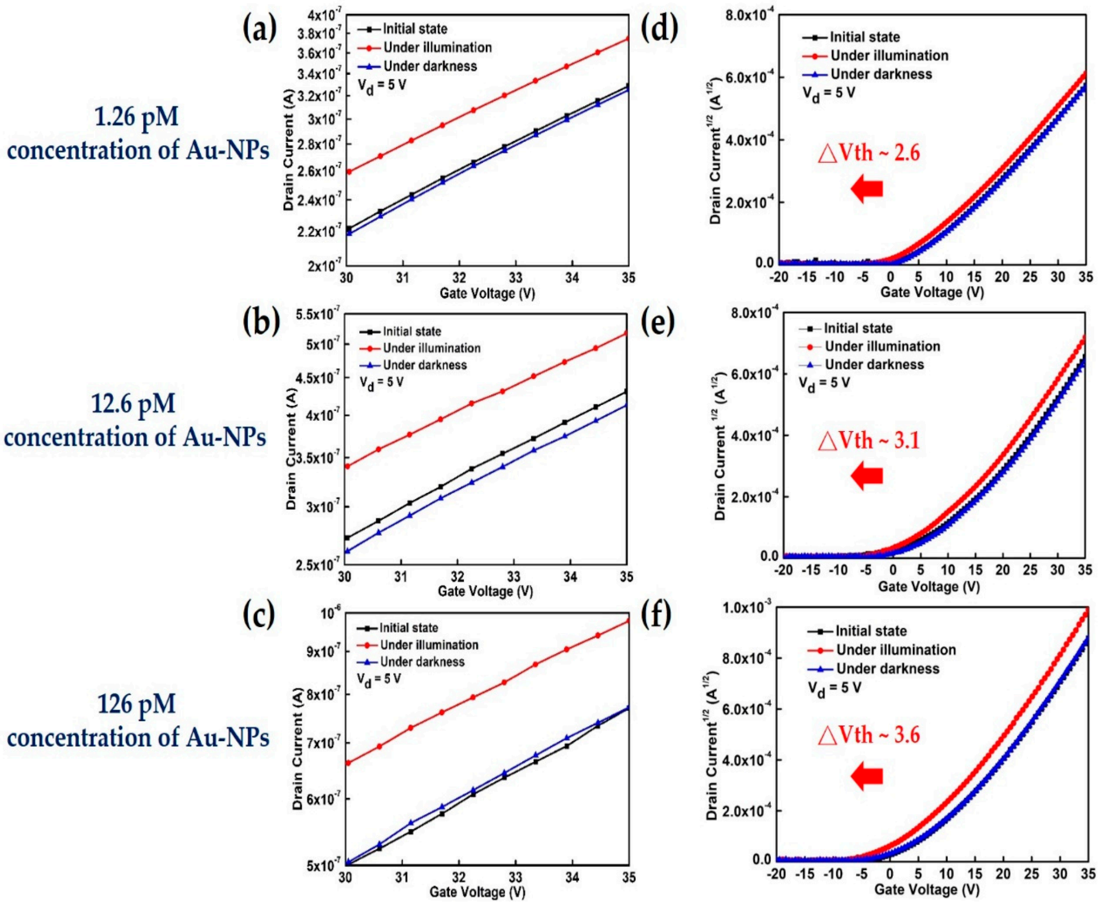

Figure S3. The typical transfer I_{DS}-V_{GS} characteristics of amplification intervals for the saturation region of the on-current between gate voltage operation of 30–35 volts, and the devices with AuNP concentration of (**a**) 1.26, (**b**) 12.6, and (**c**) 126 pM. The square root of the drain current-gate voltage transfer characteristics curves of the devices with initial state, under illumination and without illumination; the △Vth were 2.6, 3.1, and 3.6 volts which corresponded to the concentrations of (**d**) 1.26, (**e**) 12.6, and (**f**) 126 pM, respectively.

© 2019 by the authors. Licensee MDPI, Basel, Switzerland. This article is an open access article distributed under the terms and conditions of the Creative Commons Attribution (CC BY) license (http://creativecommons.org/licenses/by/4.0/).
